# Reference database design for the automated analysis of microplastic samples based on Fourier transform infrared (FTIR) spectroscopy

**DOI:** 10.1007/s00216-018-1156-x

**Published:** 2018-07-06

**Authors:** Sebastian Primpke, Marisa Wirth, Claudia Lorenz, Gunnar Gerdts

**Affiliations:** 1 Alfred Wegener Institute Helmholtz Centre for Polar and Marine Research, Biologische Anstalt Helgoland, Kurpromenade 201, 27498 Helgoland, Germany; 2 Leibniz Institute for Baltic Sea Research Warnemünde, Seestraße 15, 18119 Rostock, Germany

**Keywords:** Microplastics, Infrared, FTIR, Imaging, Database, Spectroscopy

## Abstract

The identification of microplastics becomes increasingly challenging with decreasing particle size and increasing sample heterogeneity. The analysis of microplastic samples by Fourier transform infrared (FTIR) spectroscopy is a versatile, bias-free tool to succeed at this task. In this study, we provide an adaptable reference database, which can be applied to single-particle identification as well as methods like chemical imaging based on FTIR microscopy. The large datasets generated by chemical imaging can be further investigated by automated analysis, which does, however, require a carefully designed database. The novel database design is based on the hierarchical cluster analysis of reference spectra in the spectral range from 3600 to 1250 cm^−1^. The hereby generated database entries were optimized for the automated analysis software with defined reference datasets. The design was further tested for its customizability with additional entries. The final reference database was extensively tested on reference datasets and environmental samples. Data quality by means of correct particle identification and depiction significantly increased compared to that of previous databases, proving the applicability of the concept and highlighting the importance of this work. Our novel database provides a reference point for data comparison with future and previous microplastic studies that are based on different databases.

**Electronic supplementary material:**

The online version of this article (10.1007/s00216-018-1156-x) contains supplementary material, which is available to authorized users.

**Graphical abstract:**
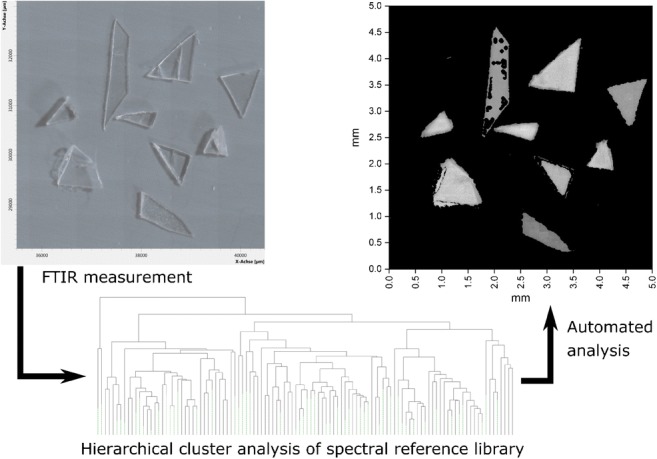
ᅟ

## Introduction

The pollution of aquatic systems with small plastic particles called microplastics (MP) [1] is an emerging topic in environmental and analytical science [2, 3]. These particles are defined as < 5 mm in size and often further divided into subcategories, e.g., large MP (5 mm–500 μm) and small MP (500–1 μm) as described by Hidalgo-Ruz et al. [4]. Two introduction pathways for MP into the environment are possible. The first is primary MP, to which the use and disposal of microbeads in cosmetic and cleaning products largely contribute [5]. The second is secondary MP formed by fragmentation of litter by mechanical or UV light-induced degradation. MP are ubiquitous in the environment [6] and their reliable monitoring is demanded within the European Marine Strategy Framework Directive (MSFD) by descriptor 10 [7]. To investigate and monitor MP pollution, it is necessary to identify the particles [2, 4]. One method is the visual identification without further chemical identification, which has a high potential of false counts. If MP are further investigated by chemical identification, up to 70% falsely assigned particles can be found [4]. Therefore, chemical identification is necessary for monitoring and different analytical methods are already in use for MP analysis.

To determine the mass of plastic within the sample, mass spectrometry is combined with pyrolysis gas chromatography (Py-GC) [8] or thermal extraction desorption gas chromatography (TED-GC) [9]. Both allow the chemical identification of the polymer types as well as the determination of mass of MP in a sample. Nonetheless, through these processes, the sample is destroyed and particle sizes and numbers cannot be calculated, which is a major drawback, for example, for ecotoxicology studies.

In contrast, spectroscopic methods like Fourier transform infrared (FTIR) and Raman spectroscopy enable the measurement of particle numbers and sizes as well as polymer identification. Both methods identify the MP polymers through their molecular vibrations in a complementary manner [10] and can be introduced into microscopic setups, which allows chemical imaging [11–13]. Single-element detectors, which are already frequently applied in MP analysis [14], were used for first setups for chemical imaging [15]. Their major drawback is the high measurement time necessary for large field sizes. The application of focal plane array (FPA) detectors enables fast measurements of large field sizes with high resolution [11], called FTIR imaging. Typically, the (in)organic matrix of environmental samples is reduced prior to measurement by chemical or enzymatic treatment and the residue concentrated onto filters [2, 16]. In earlier studies [13, 17, 18], the complete filter areas were measured followed by an analysis through integration of plastic polymer-specific band regions for the generation of false color images. The hereby pre-selected particles had to be compared via manual comparison to reference spectra, which is a time-consuming task [10, 11, 19] and prone to human bias.

Through the development of an automated analysis pipeline [20], it was shown that the expenditure of time and human bias was reduced to a minimum, while large field sizes could be measured. Further, it was found that small microplastic particles, which were previously missed in manual analysis, could now be successfully identified. The automated analysis pipeline uses spectral correlation of the raw and first derivative of vector-normalized spectra against a reference library for chemical identification. Afterwards, both results are compared and if an identical result is found, the pixel is counted as identified. By image analysis, the size and number of particles for each polymer are determined.

For all described FTIR-based analyses, the underlying database is crucial for the quality of the results. While different methods are available for data handling [20–22], commercial databases are unsuitable for these methods. Application of the automated analysis on different samples made it clear that for standardized analyses, a specialized database design is necessary to distinguish between different materials in specific spectral ranges [23–25].

In the case of the automated analysis, the spectra have to be sorted into clusters, which are necessary, as no categorization or too fine categorization would lead to errors in the assignment process. Large gaps or several small particles instead of one large particle would be assigned, falsifying the determined particle abundance derived by image analysis. Especially in a large database with several entries of similar materials, this is likely to happen, as the same material can be assigned to different database hits. For standardization of MP analysis, the database should be designed with adaptability for future research questions, while the original design can serve as a reference point for future versions and different spectral ranges and derivatives.

In this study, we present a detailed novel approach for an adaptable database design (ADD) for the automated analysis based on statistical methods followed by validation. Therefore, we investigated the typical spectral range (3600–1250 cm^−1^) for Anodisc [11] filter material regarding differences within the reference spectra by cluster analysis. By manual evaluation of the generated clusters and further validation, an initial reference library in the spectral range 3600–1250 cm^−1^ was determined for ADD and evaluated, which can serve as a basis for future database adaptations.

## Materials and methods

### FTIR measurements

To set up a general spectral database, polymer samples from different suppliers were measured via attenuated total reflection (ATR)-FTIR spectroscopy on a Bruker Tensor 27 System (Bruker Optics GmbH) with a diamond platinum ATR-unit (Bruker Optics GmbH). The spectra were recorded in absorbance mode within the range from 4000 to 400 cm^−1^ with a resolution of 4 cm^−1^ and 32 scans were co-added. Each measurement was performed in triplicate. Selected materials were additionally measured in transmission mode via a μFTIR microscope (see below) at a resolution of 8 cm^−1^ with six co-added scans.

The FTIR imaging measurements were performed on a Bruker Tensor 27 spectrometer connected to a Hyperion 3000 μFTIR microscope (Bruker Optics GmbH) equipped with a 64 × 64 FPA detector. The microscope is equipped with a × 4 lens for the collection of visual images of the sample surface and × 15 Cassegrain objectives for IR analysis. Data collection was performed with the OPUS 7.5 (Bruker Optics GmbH) software. All data shown was measured with 4 × 4 binning at a resolution of 8 cm^−1^ with six co-added scans in accordance with literature [11]. The minimum detectable particle size with these parameters was 11 × 11 μm.

### Spectral database design

The recorded ATR spectra were processed using the OPUS 7.5 software. Three spectra for each sample were averaged and an infobox was created containing sample name, abbreviation, supplier, source ID, form, color, and method. The spectra were baseline corrected using the concave rubberband correction with 10 iterations and 64 baseline points. In the case of black material, the spectra were subjected to an extended ATR correction beforehand. For entries based on transmission FTIR measurements, 20 single spectra were isolated from each dataset and afterwards treated as described above. However, a straight line was generated in the wavenumber range of 2420–2200 cm^−1^ to exclude the CO_2_ band. All spectra were made compatible so they contain the same number of wavenumber datapoints in the considered spectral range (x axis). Spectra with a low signal-to-noise ratio were excluded afterwards. The combined data is further provided as a Microsoft Excel Sheet (ESM_2.xslx) within the Electronic Supplementary Material (ESM). Samples of different types of polymer-based fibers as well as of different plant types and all animal furs were received from the Bremer Faserinstitut in Germany.

### Automated analysis and image analysis

The automated analysis and image analysis were conducted as described in previous work [20]. Briefly, all spectral analyses were performed on HP KP719AV computers equipped with an Intel© Core 2 Duo™ processor, 8-GB RAM, AMD Radeon HD 5450 graphic card, extra USB3.0 controller card, and a SANDISK Extreme 64-GB USB stick. The library searches were performed through a macro within the OPUS 7.2 software.

For image analysis, the raw data was analyzed by Python Script and SimpleITK [26, 27] functions using Anaconda (Anaconda, Inc.) and Spyder on a HP Z400 workstation with an Intel© Core Xeon W3550 CPU, 12-GB RAM, NVIDIA Quadro FX 1800 graphic card, and an additional CSL PCI Express Card USB3.0 controller. The results of the image analysis were further investigated using OriginPro2017G (OriginLab Corporation).

### Cluster analysis with PRIMER 6

To generate clusters, spectra were subjected to a hierarchical cluster analysis using the Primer 6 software equipped with the Permanova+ package (PRIMER-E). For this, all negative values in the spectra were set to 0. To exclude effects from different concentrations and varying contacts between diamond crystal and material during the ATR measurement, all data was normalized to percentage. For the analysis, the Hellinger distance of the different spectra was calculated and subsequently subjected to cluster analysis.

For further investigations of the cluster analysis, the similarity profile (SIMPROF) routine, a permutation procedure that tests for the presence of sample groups, was used [28]. When applying it to the analysis of dendrograms generated via hierarchical cluster analysis, it can provide stopping rules for further fractionation of samples into subgroups.

### Reference samples

Ref7P: For preparation of a reference sample with known content, synthetic polymers as well as natural materials (seven in total, see Table S1, ESM_3.pdf) with a size range from approx. 150 μm down to a few microns were mixed. In a glass bottle with a ground joint and stopper, each material was given into MilliQ (30 mL, 0.22 μm, Merck Millipore) and the spatula was washed afterwards thrice with 30% ethanol (3 × 1 mL, filtered over 0.2 μm) each time. A Teflon-coated stirring bar was added. Prior to filtration, the mixture was stirred for 30 min on a magnetic stirrer and 1 mL of the mixture was filtered onto an Anodisc filter (0.2 μm, GE Whatman). The filter was washed with 30% ethanol (5 mL) and dried for 24 h at 30 °C. The sample was placed under the μFTIR microscope and measured via FTIR imaging in the range of 3600–1250 cm^−1^.

Reference filters RefA to RefD: For each reference filter (see Tables S2–S5 and Figs. S1–S4 for details, ESM_3.pdf), small particles were either produced by cutting from polymer foils or fine-grinded polymer samples were directly applied. In each case, up to 11 materials were placed manually under a stereomicroscope (SZX16, Olympus) onto an Anodisc (0.2 μm) filter. The position and shape of the particles were determined via an overview image prior to FTIR measurement. The FTIR imaging measurements were performed in a spectral range of 3600–1250 cm^−1^.

RefEnv1: To make the results comparable to those of our previous study [20], the therein analyzed environmental sample H18_21 was used as reference for the automated analysis. As the original measurement was only conducted in the spectral range of 3200–1250 cm^−1^, the sample was re-measured via FTIR imaging with a range of 3600–1250 cm^−1^, as described above.

Environmental sample RefEnv2: The environmental sample was chosen from a previous study [25] of samples from waste water treatment plants. It was collected at waste water treatment plant Oldenburg on 13 August 2015 in front of a post-filtration unit. The (in)organic matrix was removed via enzymatic digestion [16, 17]. For comparison, the sample was re-measured in the spectral range of 3600–1250 cm^−1^ (original study 3200–1250 cm^−1^) [25], as described above. Prior to image analysis, the data belonging to the polypropylene support of the Anodisc filter was removed.

All reference datasets are available in the ESM as JCAMP-Dx files .

### Spectral validation

To determine the data quality, the identified spectra were additionally analyzed manually by expert knowledge. For this, the spectra were opened with the OPUS 7.5 software and compared to the assigned reference spectra. The measured spectra were visually compared to the assigned reference spectra regarding the presence/absence of essential and additional bands. The categorization of the data quality was performed in accordance with literature [20]. Each spectrum was labeled with a number of either 1, 0.75, 0.5, 0.25, or 0.01 in dependence of the number of minor or major differences: 1 = no difference, 0.75 = one minor difference, 0.5 = two minor differences, 0.25 = three minor or one major difference, 0.01 ≥ three minor differences, or > one major difference.

## Results and discussion

### Data quality and modifications

For the design of the ADD, it has to be considered that the achieved spectral quality for FTIR imaging is generally lower than that for ATR measurements. Therefore, ATR spectra that feature only minor differences needed to be grouped into clusters. After the collection of all database spectra, it was found that the ATR data showed a systematic artifact of the crystal in the region from 2475 to 1970 cm^−1^. The influence of the artifact was tested with a small dataset of eight polymer types including low-density polyethylene (LDPE) and high-density polyethylene (HDPE). Both materials only showed small differences between the spectra (see Fig. S5, ESM_3.pdf). When the artifact was replaced with a straight line (see Fig. S6, ESM_3.pdf), it was possible to distinguish between HDPE and LDPE, while it was impossible if the artifact was present. All further materials could be well separated by cluster analysis independent of the artifact. As no decisive information can be measured within the region of the artifact by ATR-FTIR [29], the data in this region was replaced with a straight line for the subsequent statistical cluster analysis.

### Cluster generation

First, it was evaluated whether SIMPROF is suitable to automatically generate clusters from the obtained dendrogram by calculating which spectra belong to the same statistical subgroup. In Fig. S7 (ESM_3.pdf), the red dashed lines in the dendrogram show clusters that SIMPROF determined as belonging to the same subgroup. Hence, according to SIMPROF, most spectra in Fig. S7 (ESM_3.pdf) belong to different subgroups and clusters respectively. When the significance level was lowered to 1% or increased to 10%, the findings did not change significantly. Apparently, SIMPROF is not a suitable method to determine the clusters. Even though the spectra all belong to different subgroups according to SIMPROF, they cannot be left as individual spectra for the automated analysis, as explained above.

**Fig. 1:**
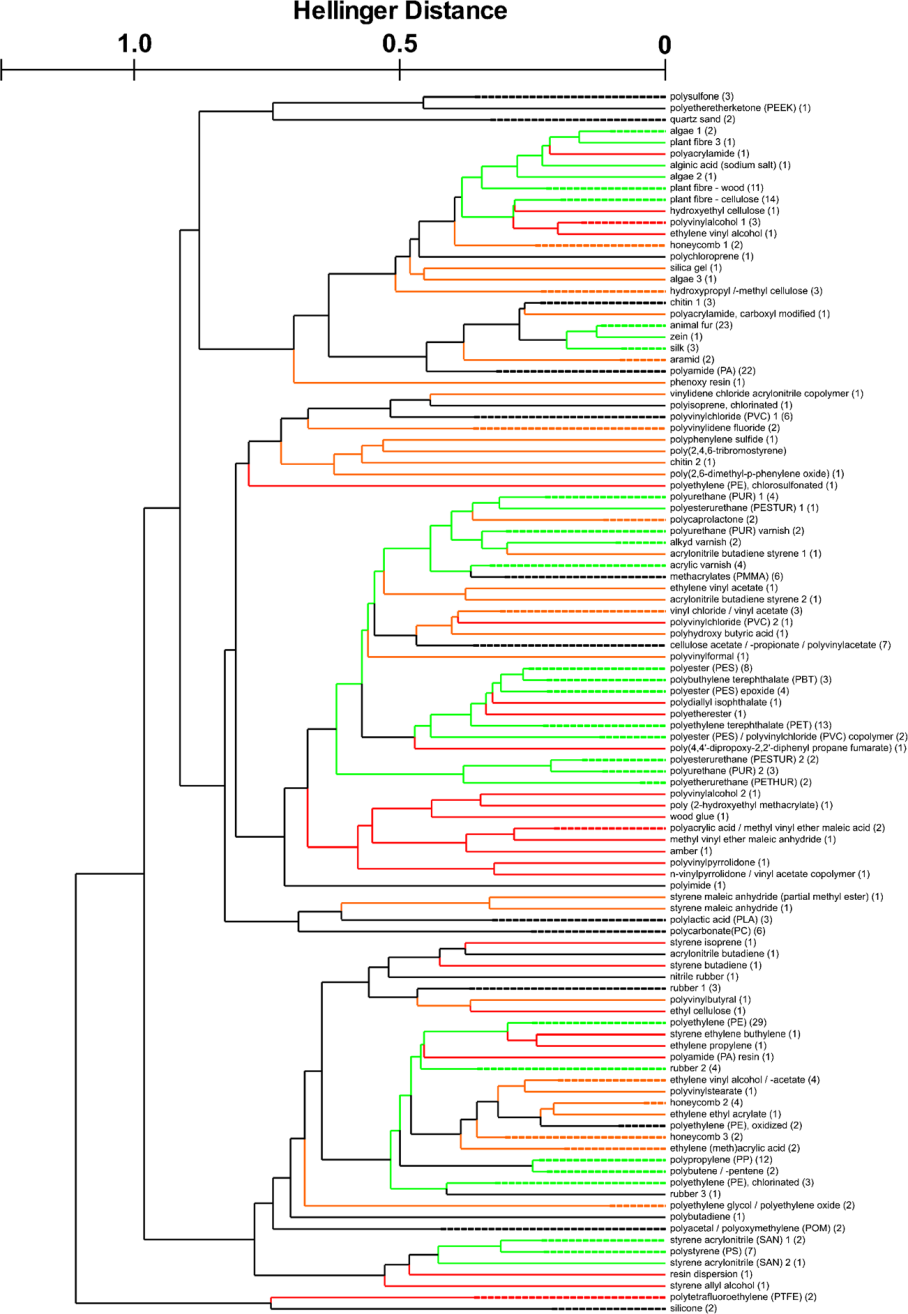
Dendrogram of manually generated clusters. For lucidity, the spectra were grouped and the number of contained spectra written in brackets behind the cluster name. All merged clusters (see text for details) were connected by green lines; for all later excluded clusters, the lines are marked red (reduction of clusters) and orange (cluster categories)

The alternative was the manual generation of clusters. In this case, spectra were grouped into clusters if (1) they were positioned on the same branch of the dendrogram and (2) the spectra were identical or showed only minor differences by expert knowledge. Spectra of the same polymer were also grouped when greater differences between the spectra were present but only if (1) was still fulfilled. Consequently, clusters had to be generated manually based on expert knowledge. Figure 1 shows the dendrogram that was obtained when 319%-normalized spectra from the ATR database were subjected to a hierarchical cluster analysis. In total, 107 clusters were generated manually. They usually consisted of more than five and up to 29 spectra. In contrast, 56 clusters only contained one spectrum. The latter ones were spectra of rather unconventional polymers and other substances, of which only one sample could be provided. Numbers were assigned to all generated clusters and a library with 107 database entries was created.

### Cluster optimization by reduction of clusters

In a first approach, reference samples RefEnv1 and Ref7P were applied for performance tests of the initial library. The analysis results were compared against the already-validated results obtained with the database used in Primpke et al. [20]. Required modifications were evident when (1) individual particles consisted of different database entries or (2) expected particles did not get detected at all. The first case was caused when the different pre-processing routines of the automated analysis yielded the same database entry but found different entries on the same particle. When the different routines yielded different database entries and no match was found at all, the second case ensued.

**Fig. 2:**
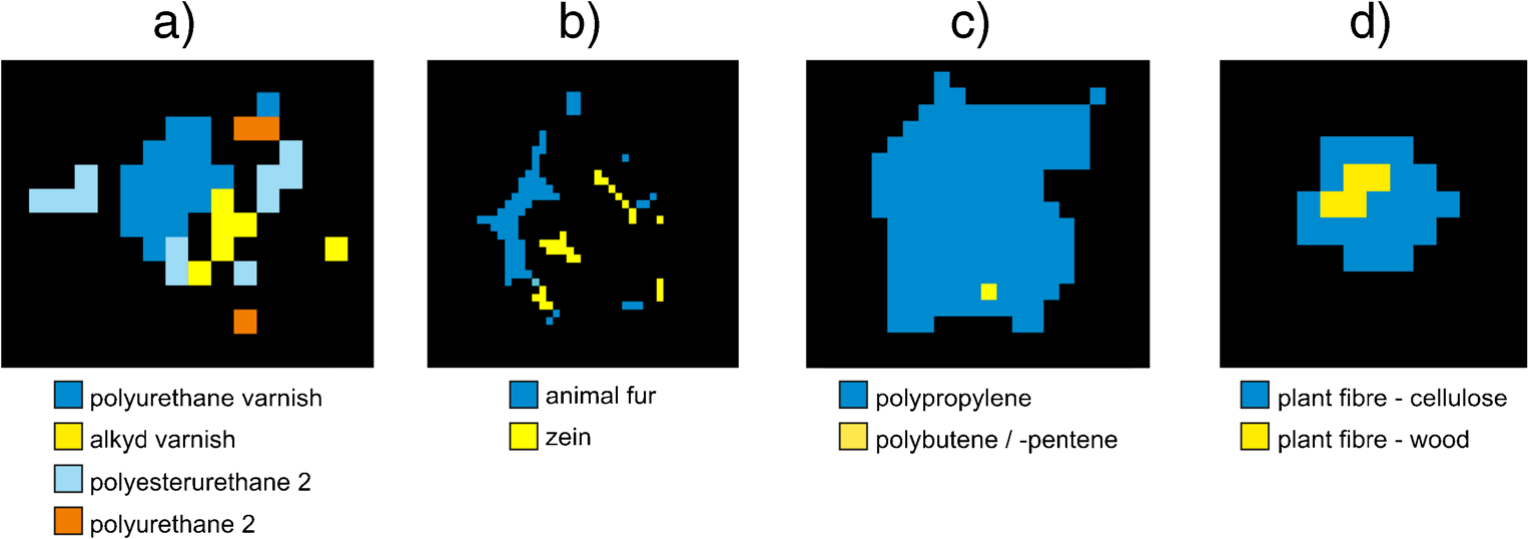
Image analysis pictures of single particles with different assigned database entries

Figure 2 shows image analysis pictures that include examples of single particles from filters RefEnv1 and Ref7P, which showed matches with two or more different database entries. The polyurethane (PUR) particle from Fig. 2a gave matches with four different clusters: PUR 2, PUR varnish, polyester urethane (PESTUR) 2, and alkyd varnish. This shows that within the given spectral region and quality, it is not possible to distinguish between polyurethanes and acrylic or alkyd varnishes. This is understandable, as the urethane group structurally resembles the ester groups present in alkyd varnishes or acrylic polymers. In a similar manner, it was not possible to separate animal fur (keratins) from zein, a protein material extracted from corn (Fig. 2b). Figure 2c, d shows further examples for polypropylene (PP), where one pixel was assigned to polybutene/polypentene and a cellulose particle, where it was impossible to distinguish between cellulose from different plant sources. All clusters that were found to interfere with others were checked for structurally similar substances and merged at this step.

Based on the information gathered via the analysis of data from filters RefA, RefB, RefEnv1, and Ref7P, a number of similar changes were made, which are summarized in Fig. 1, marked in red. Nonetheless, the overall approach of evaluating the whole set of clusters at once and trying to identify all interferences was found to be unsuitable. A number of problems could be correctly identified and solved, but it became more and more evident that a large amount of modifications was necessary to create an applicable database. Hence, evaluating the full set of clusters proved to be too complex and time consuming. To further investigate the clustering for ADD, the approach was changed at this point.

### Cluster optimization based on cluster categories

In order to find a more suitable approach, the remaining 58 clusters were sorted into four categories according to their importance for microplastic analysis. Aspects like produced polymer amounts per year, water solubility, fields of application, and, in relation to that, the expected abundance in environmental MP samples were considered for the categories. The full list of remaining clusters and their assigned categories is provided in the ESM (see Table S6, ESM_3.pdf). The first category contained clusters with the most abundant plastic polymers (polyethylene (PE)/rubber, PP, polystyrene/styrene-acrylonitrile (PS/SAN), polycarbonate (PC), polyamide (PA), polyvinylchloride (PVC), polyester/polyethylene terephthalate/polybutylene terephthalate (PES/PET/PBT) and PUR/varnish), silicone, and three common natural substances: cellulose, animal fur, and quartz sand. These materials were categorized as “very important” for microplastic analysis and used for a basic library. For the verification process, the reference samples RefA to RefD were used, which consisted of materials of the different remaining clusters. This basic library was verified and all suitable clusters from categories 2 (“important”) and 3 (“less important”) were introduced stepwise into the library. The clusters from category 4 were marked “not important” for microplastic analysis at this stage and were excluded. During the process, it was evaluated for any added cluster whether all reference particles were identified, whether they were assigned the correct database entry, and whether they disturbed the assignment of any other particle.

**Fig. 3:**
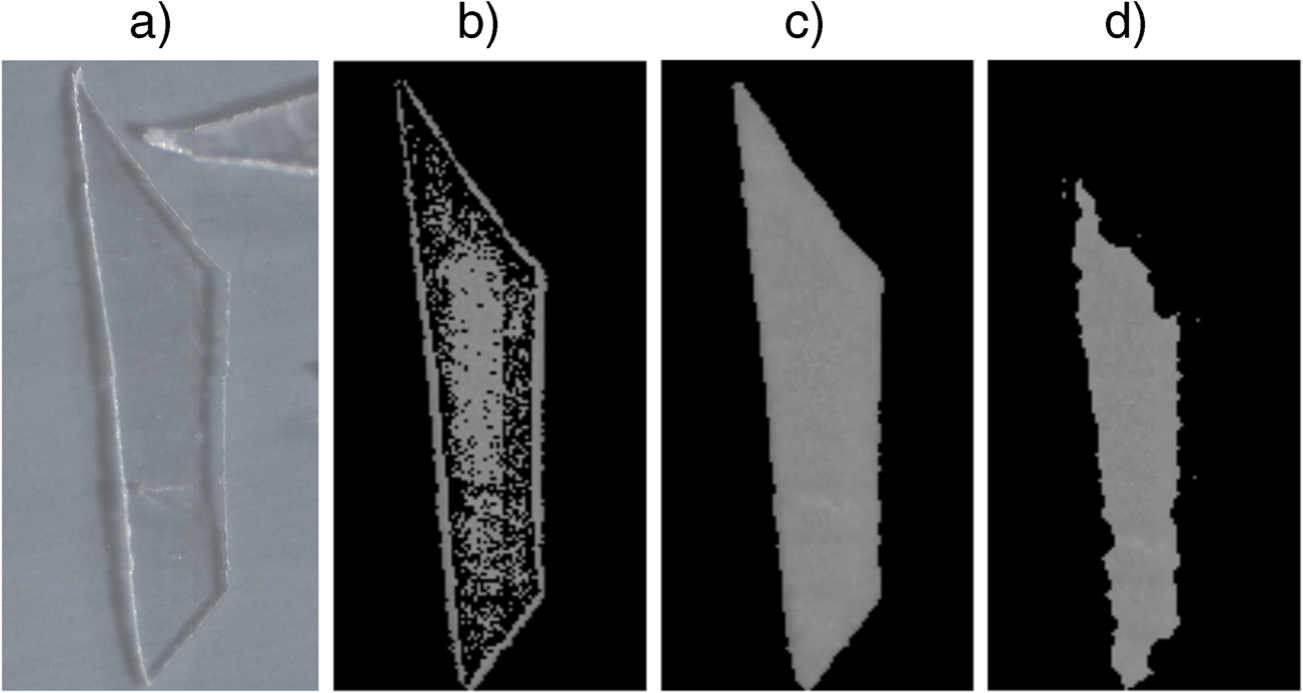
Photograph (**a**), unclosed image (**b**), and closed image (**c**) of a piece of cellulose foil. Closed image (**d**) of the same particle when silica gel is present in the adaptable database design

Figure 3 illustrates how interferences with other substances were detected. It depicts the closed image of the same cellulose particle in absence/presence of the cluster silica gel within the library (Fig. 3a–d). It is evident that with silica in the database, the edges of the particle were not detected anymore, and thus the depiction of the particle was smaller. All observed interferences were evaluated in a similar manner. However, the benefit of having a substance in the database was always weighed against the deficit that was caused by the interference. For example, “chitin 1” was found to slightly hinder the detection of cellulose but since it is a very common component of marine samples, it was kept in the library nonetheless.

All changes that were made during optimization based on cluster categories are summarized in Fig. 1, marked in orange. Upon examining the dendrogram in Fig. 1, it was striking that not all mergings of clusters that were performed during the optimization process were in unison with the structure of the dendrogram. For example, the PUR/varnish cluster consisted of two groups of clusters, which were located on different branches of the dendrogram. This showed that, while the cluster analysis was a helpful tool to sort the spectra, it was not capable of completely predicting the necessary clusters for the automated analysis. This underlines the fact that the conducted cluster optimization process was vital for the development of a functioning ADD.

During the optimization process, 20 clusters of in total 27 could be verified without constraints. To test the performance of this preliminary database, obtained results from the analysis of RefEnv1 were manually reanalyzed by expert knowledge. The data quality determination was performed as described previously in literature [20]. It was discovered that the clusters PC, polymethyl methacrylate (PMMA), polysulfone (PPSU), PS/SAN, PA, and PVC were assigned correctly within the 95% confidence interval. For PP, polyacetal/polyoxymethylene (POM), and PUR/varnish, error values between 10 and 50% were found. On the contrary, the clusters PE/rubber and silicone yielded a high number of false assignments at this stage. This showed that the ADD still required improvement. While the cluster silicone was removed, the re-separation of the PE/rubber cluster into the separate PE, chlorinated PE, and rubber 2 clusters substantially reduced the amount of misassignments and improved particle identification and depiction. A second change that became evident from the manual reanalysis was the merging of the acrylates and PUR/varnish cluster. These materials could not be distinguished from one another in the considered spectral region. No further improvements regarding the results for filters RefA to RefD could be achieved (see Fig. S8, ESM_3.pdf).

### Optimization with transmission FTIR data

While the ATR spectra yielded a suitable database for polymer identification, larger particles were often not targeted well. This is caused if total absorbance occurs during transmission measurements. If one datapoint reaches the limit of detection, the measured values will be independent of the rest of the spectrum and characteristic bands get lost. Based on filters RefA to RefD, the respective transmission spectra were collected and added to the distinct clusters manually without further analysis. This further allowed the reintroduction of the clusters polycaprolactone and ethylene-vinyl-acetate (EVA). All materials introduced as transmission FTIR data are summarized in Table S7 (ESM_3.pdf).

Afterwards, the particle identification of reference samples RefA to RefD (see Fig. S9, ESM_3.pdf) improved and most particles could be identified. In conclusion, the introduction of transmission FTIR data in ADD was found to be a necessary database extension.

### Introduction of new materials into ADD

As a last step for the setup of ADD, it was exemplarily investigated how to introduce further materials into the database. In a recent study [24], large amounts of black particles were found in deep sea sediments, which were presumed to consist of coal. To include these new materials, six spectra of coal (charcoal and conventional coal) were measured via ATR-FTIR and the data was handled as described above. After extending the dataset, an analogous cluster analysis was performed.

The coal spectra were included in the dendrogram as new clusters (see Fig. S10, ESM_3.pdf), while the overall dendrogram structure did not change significantly. The new dataset was manually binned into two new clusters for the automated analysis (charcoal, 2 spectra and coal, 4 spectra) afterwards. This process highlights the ability to add new spectra/materials to the existing ADD by a combination of cluster analysis and manual clustering. With this data included, the final reference design for ADD was determined with 32 clusters (see Table 1) and is available in the ESM (see ESM_5.xslx).

**Table 1:** Polymer clusters derived for the adaptable database design for the automated analysis via FTIR imaging including their cluster number for image analysis and number of spectra assigned

Cluster name	Cluster number adaptable database design	Number of contained spectra
Polyethylene	1	30
Polyethylene oxidized	2	2
Polyethylene-chlorinated	3	3
Polypropylene	4	15
Polystyrene	5	11
Polycarbonate	6	7
Polyamide	7	23
Polyvinylchloride	8	6
Cellulose chemical modified	9	8
Nitrile rubber	10	2
Polyester	11	31
Acrylates/polyurethanes/varnish	12	27
Animal fur	13	27
Plant fibers	14	33
Sand	15	2
Polysulfone	16	4
Polyetheretherketone	17	2
Polychloroprene	18	2
Chitin	19	3
Polyisoprene chlorinated	20	1
Polylactic acid	21	4
Polycaprolactone	22	3
Ethylene-vinyl-acetate	23	3
Polyimide	24	2
Polyoxymethylene	25	3
Polybutadiene	26	1
Acrylonitrile-butadiene	27	2
Rubber type 1	28	3
Rubber type 2	29	1
Charcoal	30	2
Coal	31	4
Rubber type 3	32	3

### Performance of the ADD

**Fig. 4:**
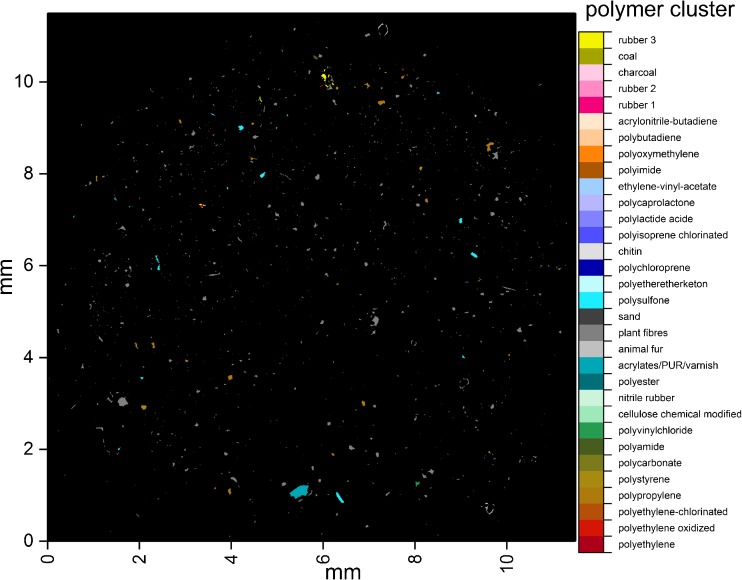
Polymer-type-dependent false color image of the sample RefEnv1 after automated analysis with the adaptable database design

The overall performance of the ADD was benchmarked against two reference samples (RefEnv1 and Ref7P) and an environmental sample (RefEnv2). Results from the analysis of RefEnv1 with the ADD are depicted in Fig. 4. For a better overview, each polymer was highlighted with a different RGB value (see Table S8 for details, ESM_3.pdf). In general, many small particles and one large particle that was assigned to the acrylates/PUR/varnish cluster were detected. High loads of plant fiber (gray), rubber type 3 (yellow), PP (brown), and PPSU (light blue) were found. The particle size distribution (see Fig. S11, ESM_3.pdf) had a maximum at the size class of 11 μm, representing 35% of the determined polymer particles, while 85% had a smaller size than 50 μm. The majority of the plastic particles were assigned to PP with 39%, PPSU with 26%, and rubber with 18%. Similar to the previous study for the automated analysis [20], the ADD was further validated by expert knowledge via manual reanalysis (see Table S9, ESM_3.pdf), and results between both studies were compared.

With the ADD, a higher amount of particles (1221 herein versus 1097 before [20]) was detected. The relative share of certain assignments increased from 82.1 to 82.8% while misassignments decreased from 3.1 to 1.6%. The general data quality, especially of PE and PVC, was several times better than that in the previous study, which shows the necessity of a well-determined database design. However, the overall number of plastic particles decreased from 733 to 195. One possible explanation would be particle loss between the measurements, which could be excluded by visual inspection of the overview images. It was found that only one prominent particle was missing and several slightly changed their position. The major difference between the present study and previous ones [20, 25] is that the range for the library search was broadened by 400 cm^−1^ to 3600–1250 cm^−1^. Diatom shells, which were still abundant in this extracted sediment sample, have a weak band (–Si–O–H bonds) in the range from 3200 to 3600 cm^−1^. It can be assumed that this band, which was present as background signal over almost the complete filter (see Fig. S12, ESM_3.pdf), hampered the identification success of plastic polymers. To test the hypothesis, the database from the previous study [20] was applied to the re-measured dataset RefEnv1 (larger wave number range). In total, 701 particles could be identified, of which 281 were made of plastic. This result is much closer to what was achieved with ADD in this study and thus confirmed the hypothesis. The second main difference between the results from previous and present database was a reduction in the amount of detected varnish particles. This is reasonable, as one particular reference varnish spectrum was not included into ADD, as the material was no longer available.

In the following, sample Ref7P was analyzed (see Figs. S13 and S14, ESM_3.pdf) to prove the ability of ADD to distinguish polymers at high sample loads with different polymers present in close proximity to each other. In total, 96.4% of the particles were assigned to the correct polymer cluster. Only in the case of copolyamide, a higher amount of misassignments (8.7%) was found, mainly to the cluster polycaprolactone. All other polymers were assigned correctly to their respective clusters for over 95% of the database hits. The results show that ADD is capable of assigning polymers even from complex mixtures and is therefore suitable for further application on environmental samples.

**Fig. 5:**
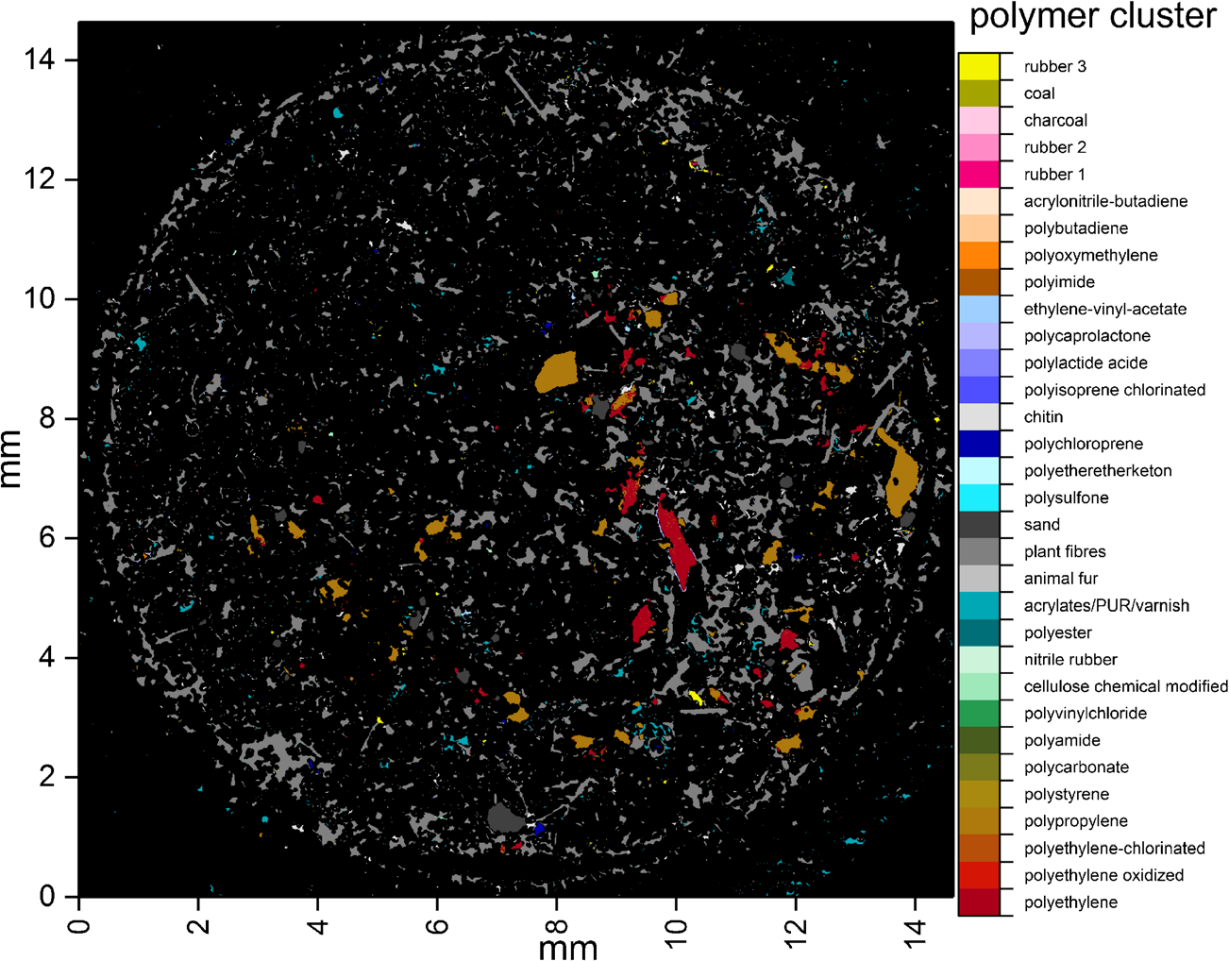
Polymer-type-dependent false color image of the sample RevEnv2 after automated analysis with the adaptable database design

**Fig. 6:**
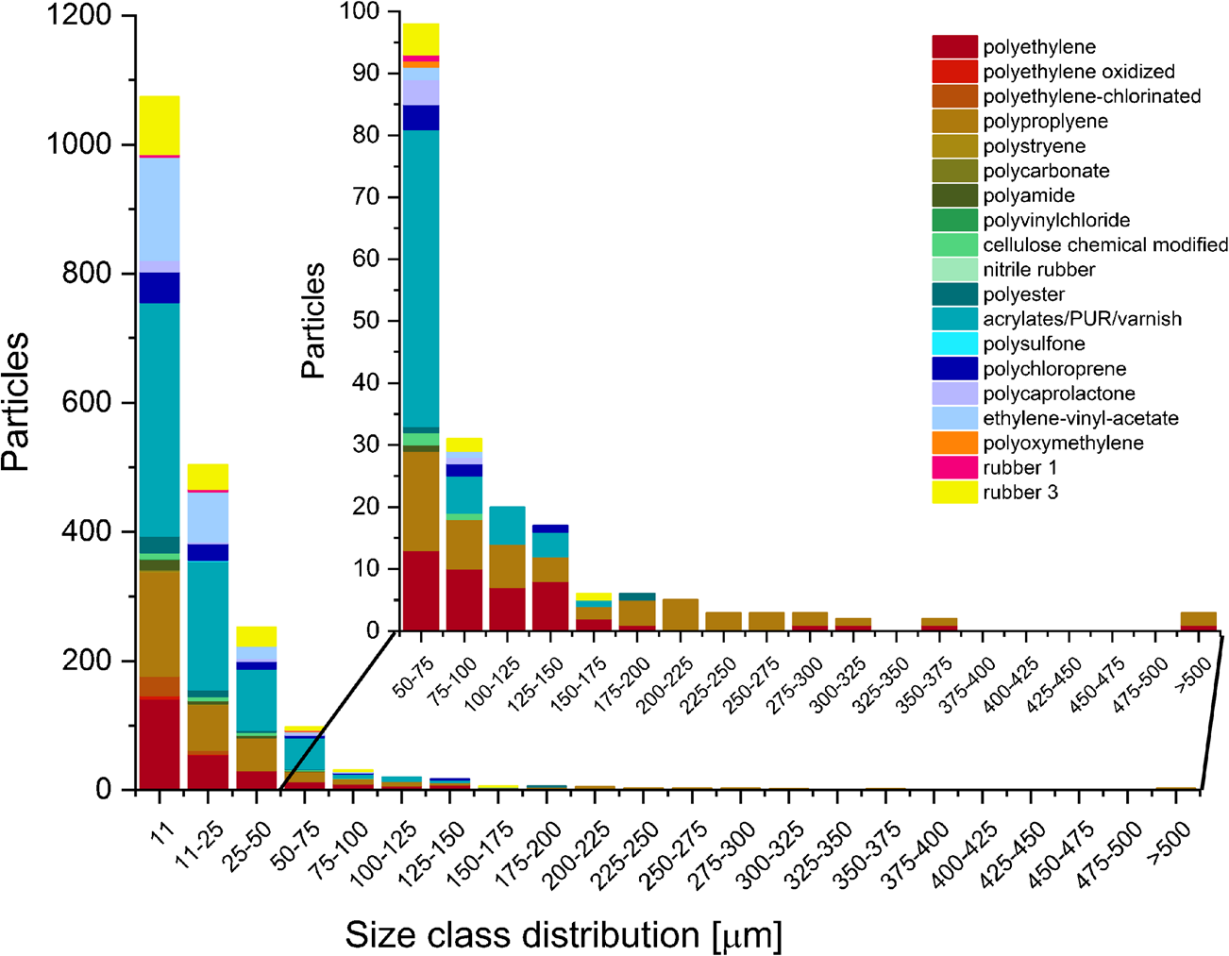
Size distribution and polymer composition for plastic particles derived via automated analysis for the sample RevEnv2. The region for particles with a size > 50 μm was highlighted for a better overview

For this, a sample of treated waste water (RefEnv2) was chosen. When applying ADD to this dataset, different types of particles, mainly PE, PP, varnish, EVA, and rubber, could be successfully identified (Figs. 5 and 6), demonstrating the high variability of polymers present in treated waste water. In the sample, 90% of plastic particles were smaller than 50 μm in size, while 53% of the overall particles were found in the smallest size class of 11 μm. The analysis of RevEnv2 highlights the performance of ADD on complex samples. Nonetheless, the previously discussed results from RefEnv1 showed that a background signal from diatom shells in extracted sediment samples can hinder polymer identification, which is currently a limitation of the method and has to be addressed during sample treatment. All in all, however, the chosen approach of combining statistical methods, expert knowledge, and manual validation proved to have produced a versatile database for the analysis of MP in environmental samples. Furthermore, the chosen approach is also suitable for Raman spectra (data not shown). First studies based on the combination of automated analysis and ADD have already been published [23–25].

## Conclusion

It could be shown that through the statistical analysis and manual clustering of reference spectra, the basis for an adaptable reference database for the analysis of MP can be provided. While the final clustering had to be based on expert knowledge, the general scheme allowed a straightforward assignment of new materials to existing entries or as entirely new entries. The generated database was benchmarked against six reference datasets and it was confirmed that the chosen setup can identify particles of various sizes and materials. Through the exemplary test on an environmental sample, it could be proven that the database is applicable to complex sample material. Moreover, the ADD can be expanded with new spectra in the future, allowing the harmonization of the FTIR analysis. In addition, by providing a reference dataset with five reference samples and an environmental sample for validation and comparison, new and old databases can be referenced to the ADD. This significantly increases the comparability of FTIR studies for past and future publications.

## Electronic supplementary material

ESM 1 (PDF 173 kb)

XLSX 2 (XLSX 8.21 mb)

ESM 3 (PDF 1.73 mb)

ESM 5 (XLSX 3.76 mb)
