# Supplementary material for: Reference database design for the automated analysis of microplastic samples based on Fourier transform infrared (FTIR) spectroscopy
Source: Anal Bioanal Chem. 2018 Jul 6;410(21):5131–41. doi: 10.1007/s00216-018-1156-x (PMC6113679; doi:10.1007/s00216-018-1156-x)
Supplement: Supplementary file 1 — (PDF 173 kb) [file 216_2018_1156_MOESM1_ESM.pdf]

Analytical and Bioanalytical Chemistry

Electronic Supplementary Material

Reference database design for the automated analysis of microplastic samples based on Fourier transform infrared (FTIR) spectroscopy

Sebastian Primpke, Marisa Wirth, Claudia Lorenz, Gunnar Gerdts

Four additional files available as 10.1007/s00216-018-1156-x
