# Supplementary material for: Reference database design for the automated analysis of microplastic samples based on Fourier transform infrared (FTIR) spectroscopy
Source: Anal Bioanal Chem. 2018 Jul 6;410(21):5131–41. doi: 10.1007/s00216-018-1156-x (PMC6113679; doi:10.1007/s00216-018-1156-x)
Supplement: Supplementary file 3 — (PDF 1.73 mb) [file 216_2018_1156_MOESM3_ESM.pdf]

Electronic supplementary material for:

Reference Database Design for the Automated Analysis of Microplastic Samples based on Fourier Transform Infrared (FTIR) Spectroscopy

S. Primpke,[a,ŧ,*] M. Wirth [a,b,ŧ], C. Lorenz,[a] and G. Gerdts[a]

[a]: Alfred-Wegener-Institute Helmholtz Centre for Polar and Marine Research, Biologische Anstalt Helgoland, Kurpromenade 201, 27498 Helgoland, Germany

[b]: Leibniz Institute for Baltic Sea Research Warnemünde, Seestraße 15, 18119 Rostock, Germany

[ŧ]: These authors contributed equally to the manuscript

Corresponding author: e-mail: sebastian.primpke@awi.de, phone number: +49 4725 819 3255

Figures:

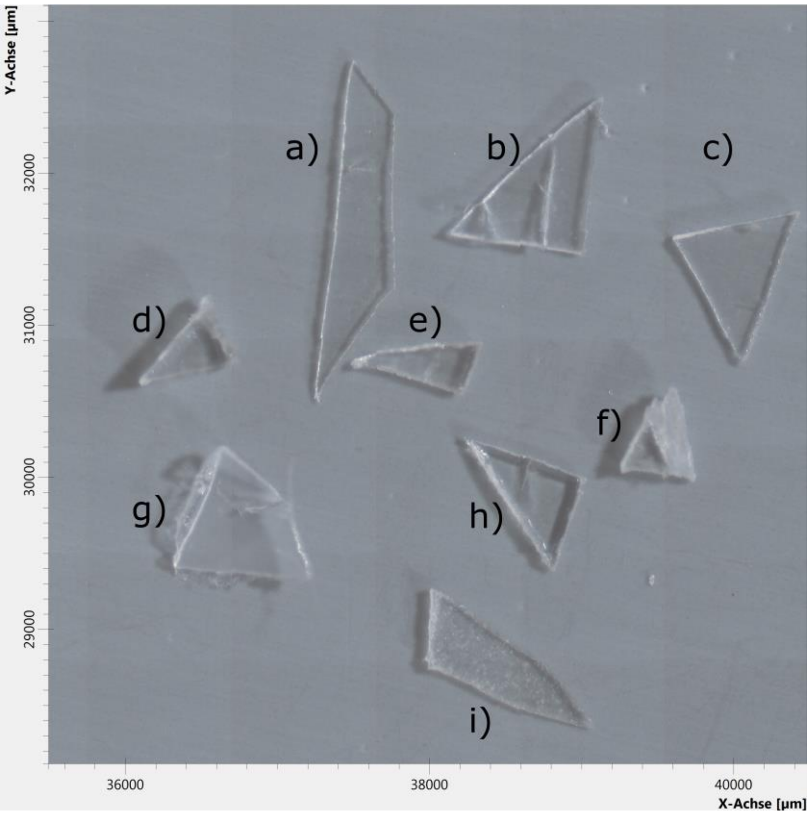

Fig. S1 Reference filter RefA for the development of the adaptable database design. a) cellulose, b) polypropylene, c) polyamide, d) polyethylene terephthalate, e) polycarbonate, f) polystyrene, g) high density polyethylene, h) polyvinylchloride, i) polyesterurethane; For detailed information see Table S2.

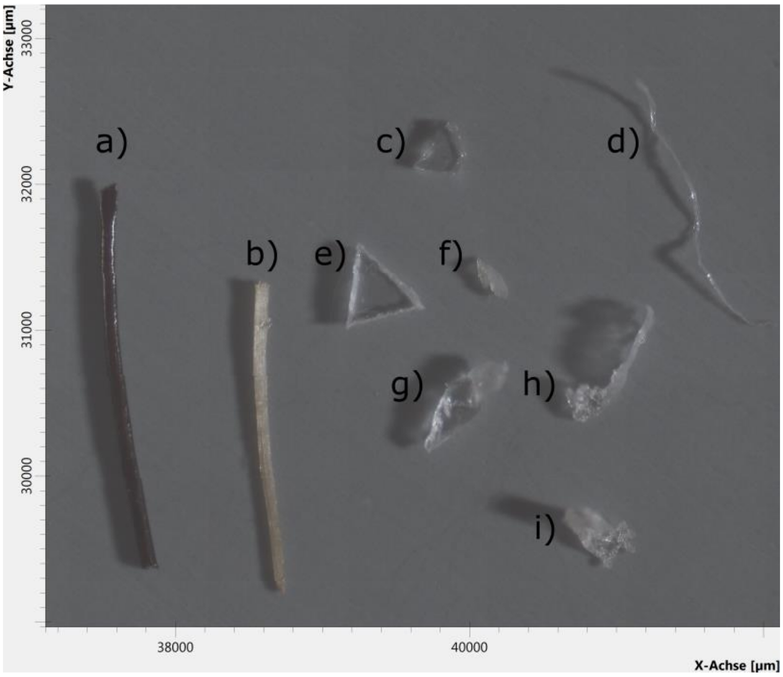

Fig. S2 Reference filter RefB for the development of the adaptable database design. a) animal fur, b) cellulose, c) methyl vinyl ether/maleic acid copolymer, d) cellulose acetate, e) styrene acrylonitrile, f) acrylonitrile butadiene, g) ethylene methacrylic acid, h) polyoxomethylene, i) polysulfone; For detailed information see Table S3.

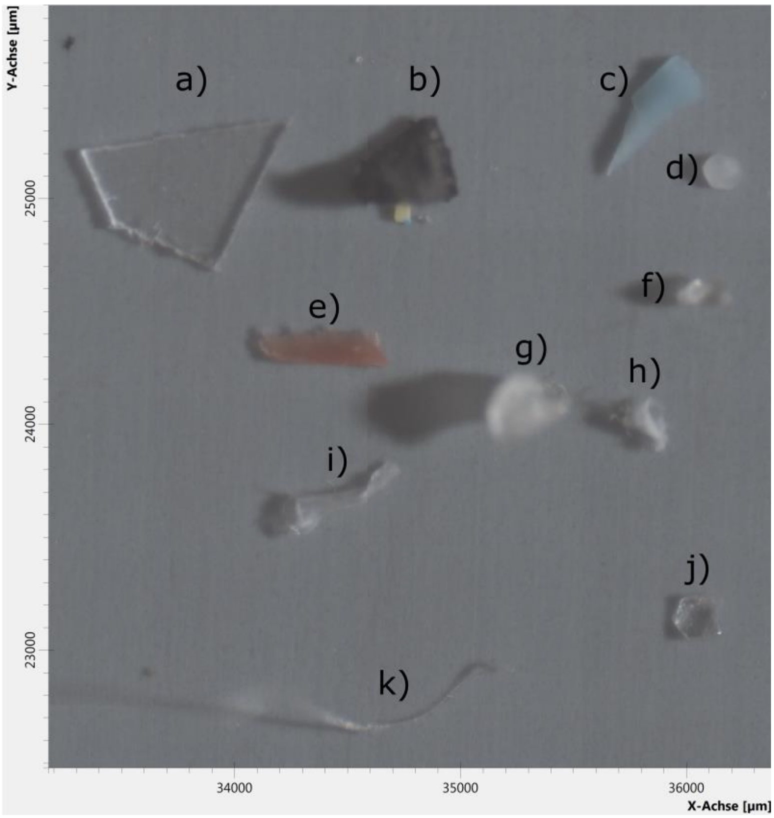

Fig. S3 Reference filter RefC for the development of the adaptable database design. a) polylactic acid, b) black rubber, c) nitrile rubber, d) polyethylene, oxidized, e) silicone, f) polychloroprene, g) chitin, h) polycaprolactone, i) sand, k) animal fur; For detailed information see Table S4.

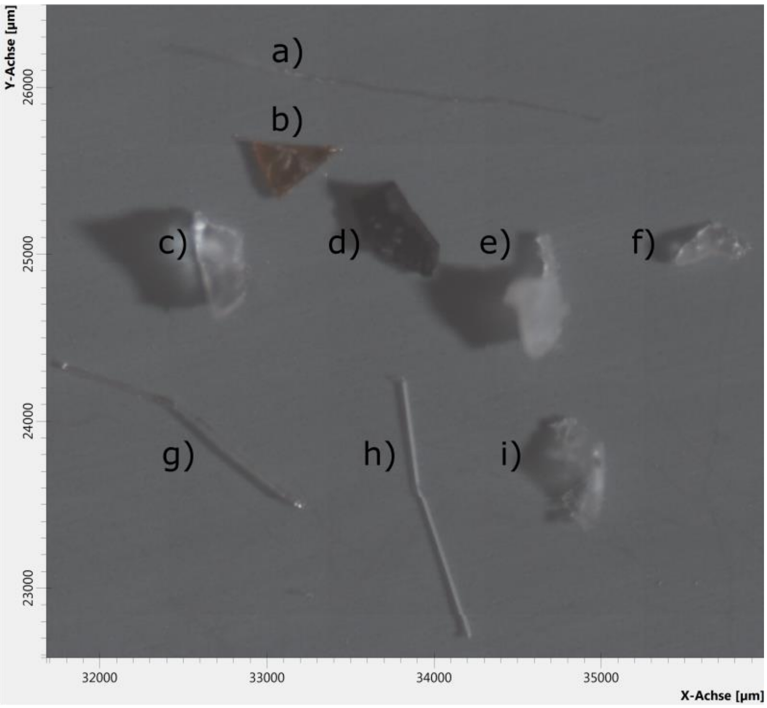

Fig. S4 Reference filter RefD for the development of the adaptable database design. a) aramid, b) polyimide, c) ethylene vinyl alcohol, ethylene vinyl acetate copolymer (40 %), d) black rubber, e) polyhydroxy butyric acid, f) polybutadiene, g) polyetheretherketone, h) polylactid acid, i) ethylene vinyl alcohol, ethylene vinyl acetate copolymer (14%),; For detailed information see Table S5.

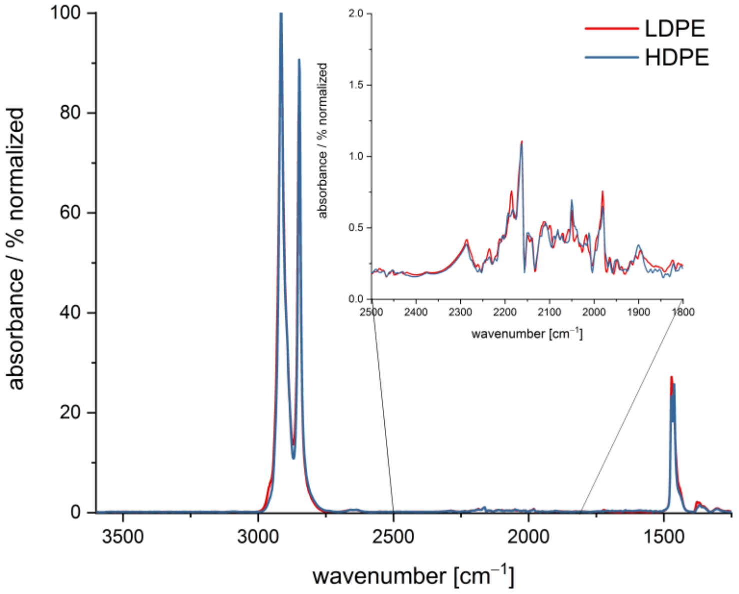

Fig. S5 Exemplary spectra for HDPE and LDPE showing the ATR artefact in the range from 2475-1970 cm^{-1} further highlighted by magnification of the data from 2500-1800 cm^{-1}.

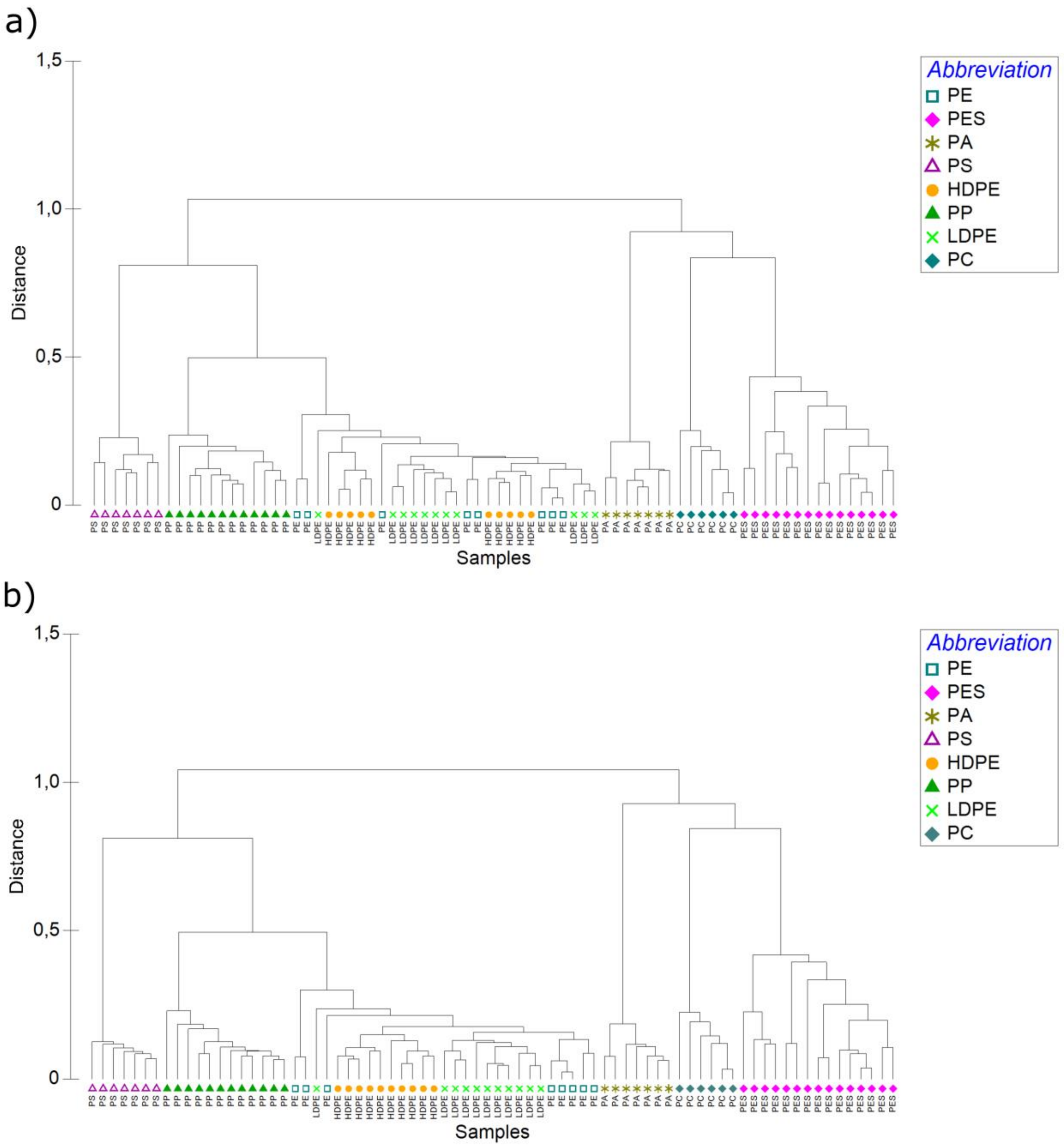

Fig. S6 A) Hierarchical cluster analysis of a selection of spectra containing the ATR – artefact between 2475-1970 cm^{-1}. B) Hierarchical cluster analysis after manipulation of the artefact to a straight line. Abbreviations: PS = polystyrene, PP = polypropylene, PE = polyethylene, LDPE = low density polyethylene, HPDE = high density polyethylene, PA = polyamide, PC = polycarbonate and PES = polyester.

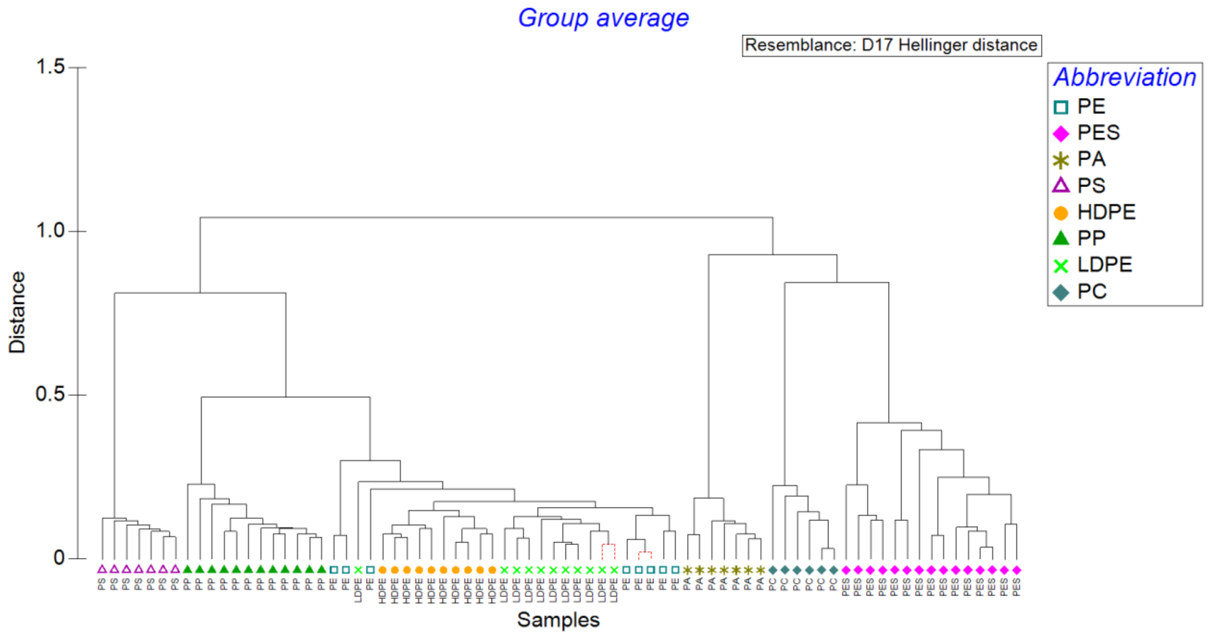

Fig. S7 SIMPROF Abbreviations: PS = polystyrene, PP = polypropylene, PE = polyethylene, LDPE = low density polyethylene, HPDE = high density polyethylene, PA = polyamide, PC = polycarbonate and PES = polyester.

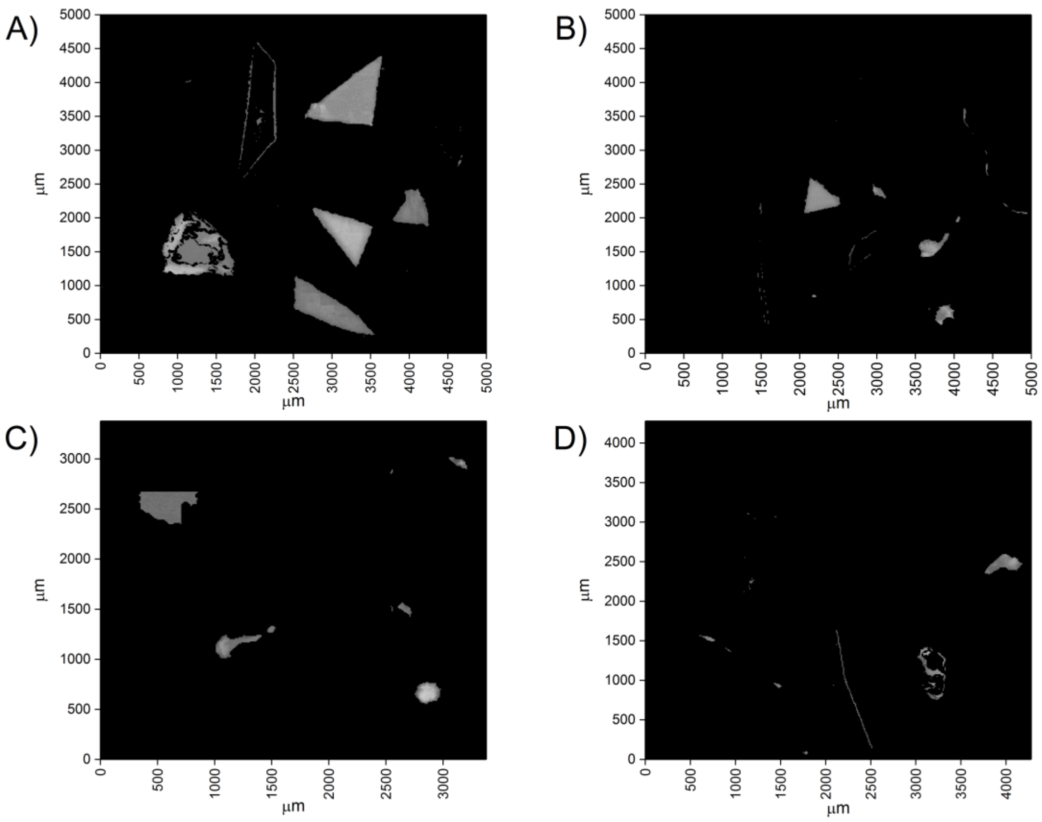

Fig. S8 Intermediate results for the application of the database determined solely by manual cluster generation of ATR-FTIR-data on the filters RefA (A), RefB (B), RefC (C) and RefD (D) with the quality factor in greyscale.

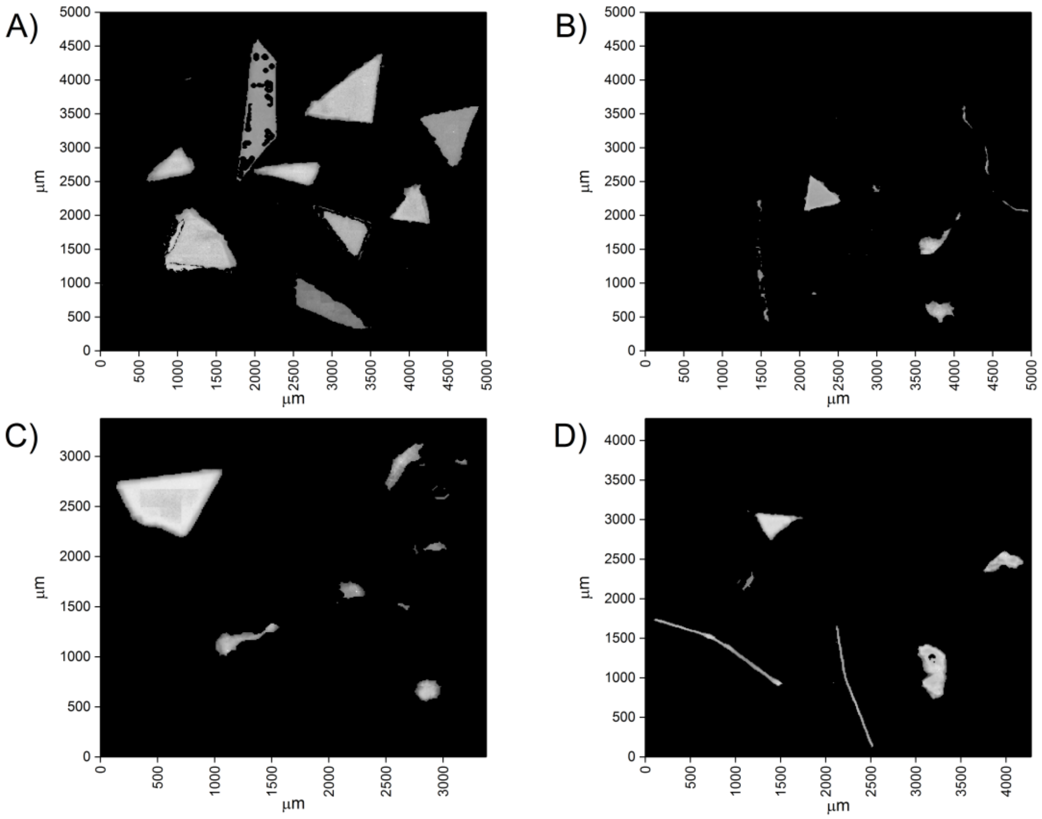

Fig. S9 Final results for the application of adaptable database design on the data of filters RefA (A), RefB (B), RefC (C) and RefD (D) with the quality factor in greyscale.

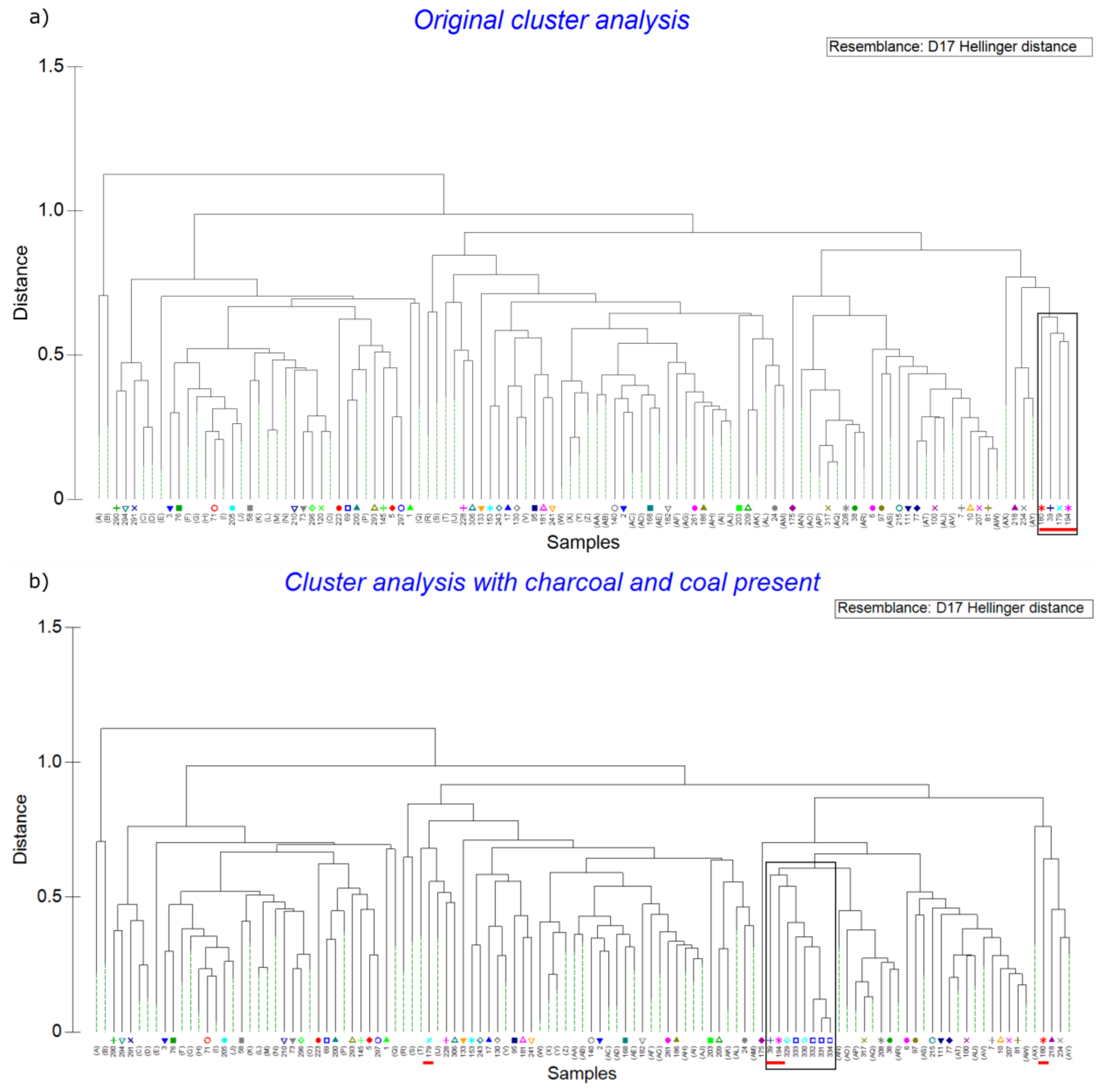

Fig. S10 a) Cluster analysis prior to the introduction of coal spectra. b) Cluster analysis after the introduction of coal spectra. The changes within the dendogram structure were highlighted by a black square and all cluster changing their position underlined by a red bar.

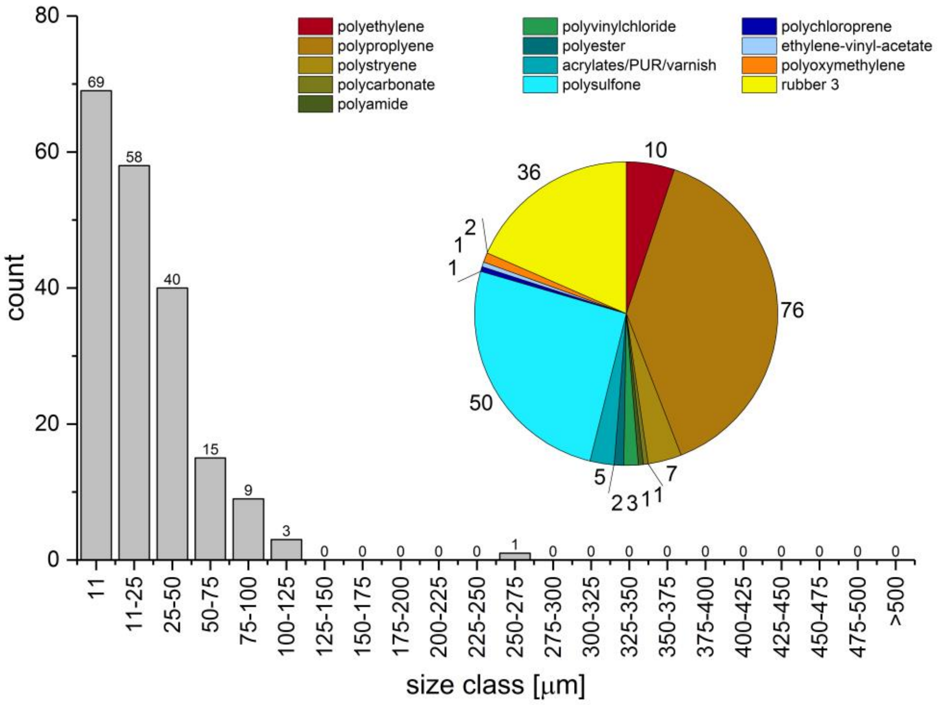

Fig. S11 Size distribution and polymer composition for plastic particles derived via automated analysis for reference sample RefEnv1.

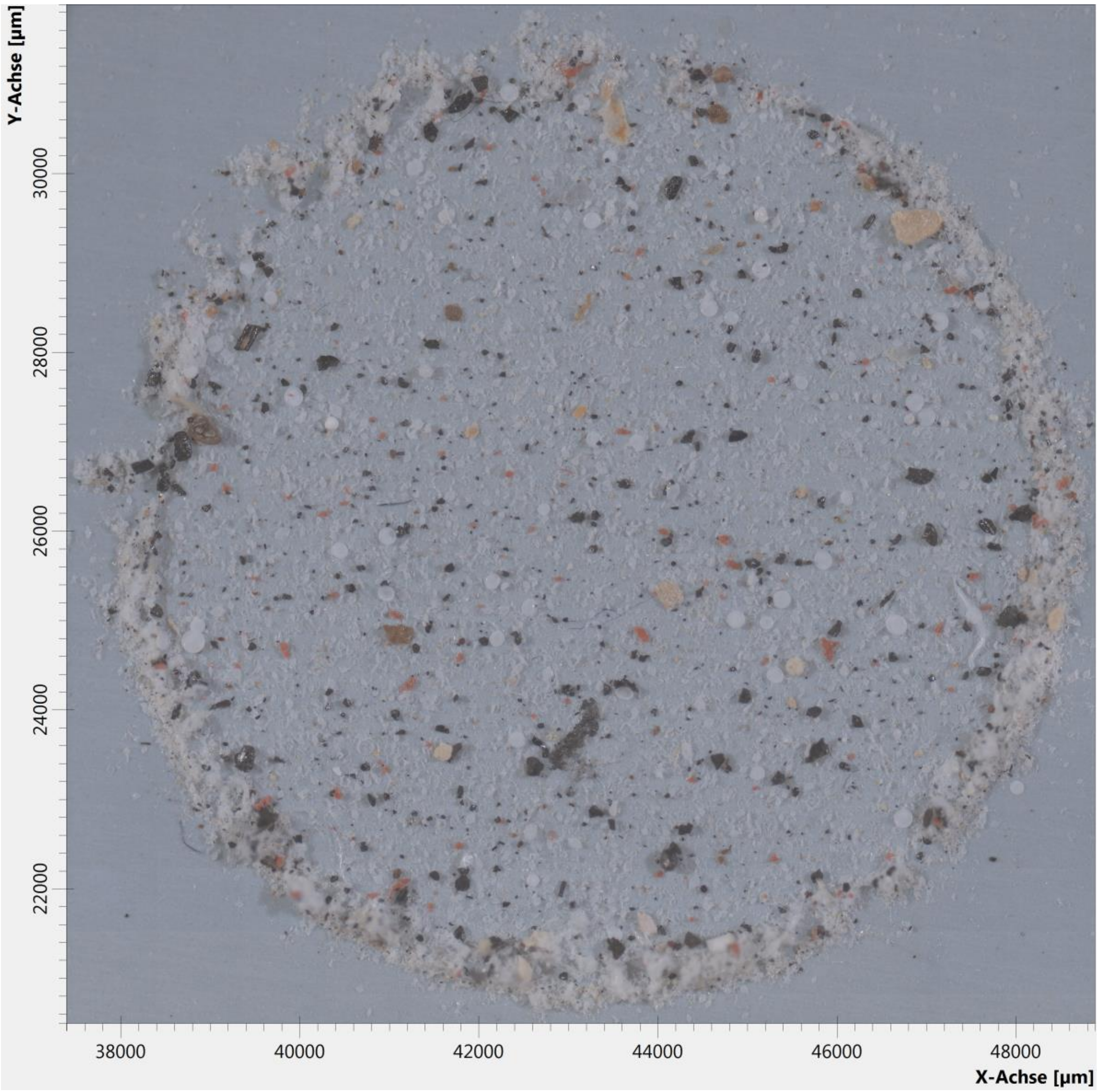

Fig. S12 Visual overview image of sample RefEnv1.

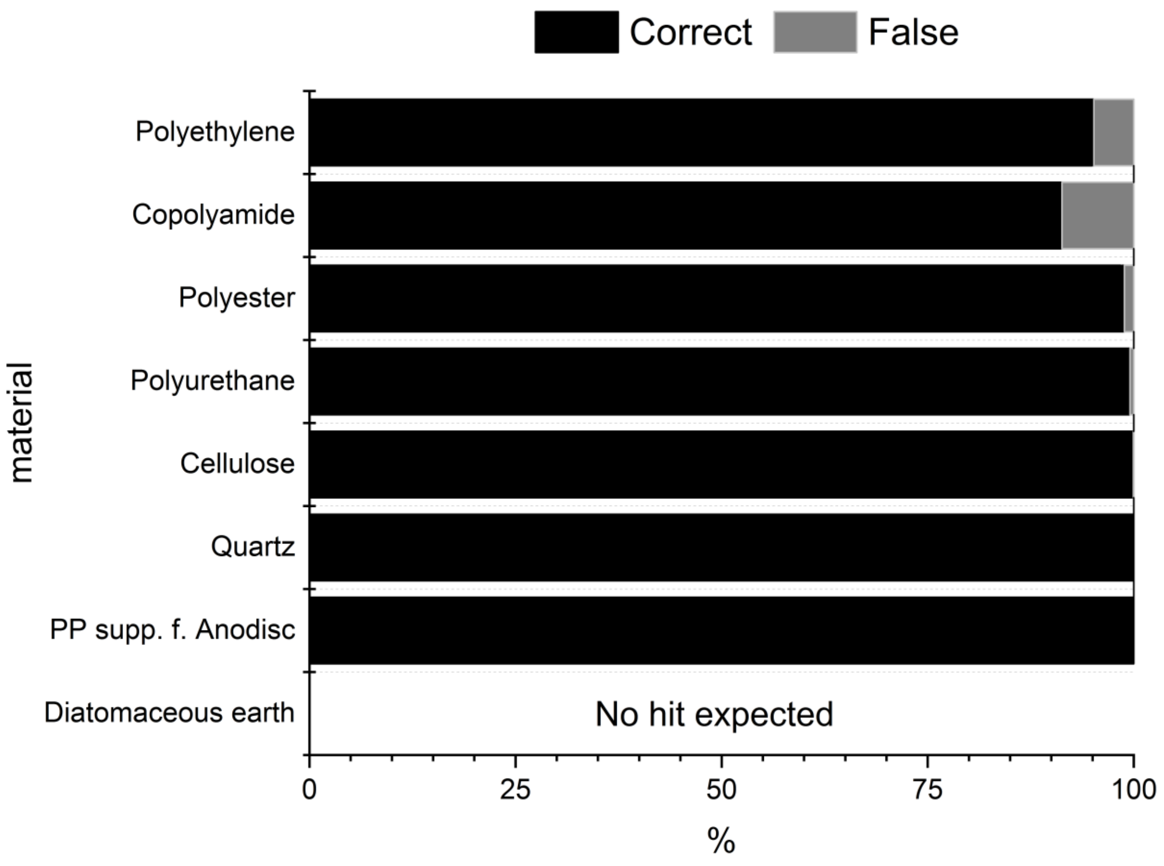

Fig. S13 Result of the analysis of Ref7P for the materials present on the Anodisc. Diatomaceous earth cannot be determined in the region from 3600-1250 cm^{-1} yet hampers the analysis of the target materials. Polypropylene (PP) was present from the support ring (supp. f.) of the Anodisc filter material at the corners of the image.

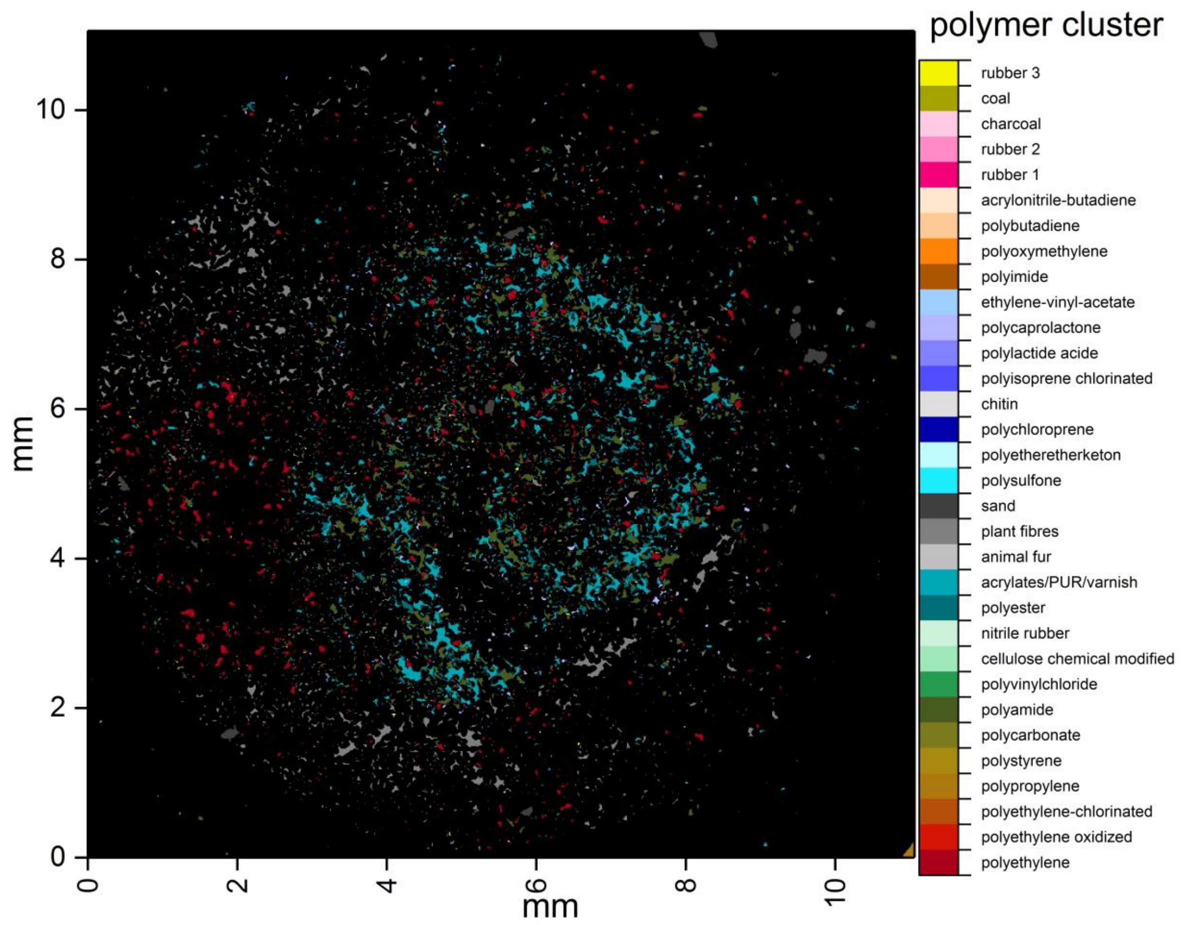

Fig. S14 False color image of Ref7P after automated analysis.

Tables:

Table S1 Polymers and natural material used for the sample Ref7P with the assigned size range and manufacturer

polymer	size	manufacturer
Low density polyethylene	0-80 µm	Schaetti AG
Copolyamide	0-80 µm	Schaetti AG
Polyester	0-80 µm	Schaetti AG
Polyurethane	0-80 µm	Schaetti AG
Quartz	Fine granular	Merck
Cellulose	Fine powder	Scientific Polymers
Diatomaceous earth	Fine powder	Sigma-Aldrich

Table S2 Materials placed on the Anodisc for sample RefA.

polymer	sample form	ATR spectrum	manufacturer	position on Filter (see Figure S1)
cellulose	foil 40µm	034	Pütz GmbH + Co. Folien KG	a)
polypropylene	foil	252	Orbita Film GmbH	b)
polyamide	foil 60 µm	158	mf-folien GmbH	c)
polyethylene terephthalate	foil 175 µm	174	Pütz GmbH + Co. Folien KG	d)
polycarbonate	foil 125µm	138	Dr. Dietrich Müller GmbH	e)
polystyrene	foil 150 µm	254	Ergo.fol norflex GmbH	f)
polyethylene, high density	foil	080	Orbita Film GmbH	g)
polyvinylchloride	foil	263	Leitz	h)
polyesterurethane	foil	256	BAYER	i)

Table S3 Materials placed on the Anodisc for sample RefB.

polymer	sample form	ATR spectrum	manufacturer	position on Filter (see Figure S2)
animal fur	fibre	266	Faserinstitut Bremen	a)
cellulose	fibre	104	Faserinstitut Bremen	b)
methyl vinyl ether / maleic acid copolymer	particle	129	Scientific Polymer Products, Inc.	c)
cellulose acetate	fibre	318	Gizeh	d)
styrene acrylonitrile	foil 90 µm	271	Ergo.fol norflex GmbH	e)
acrylonitrile butadiene	particle	005	Scientific Polymer Products, Inc.	f)
ethylene methacrylic acid	particle	072	Scientific Polymer Products, Inc.	g)
polyoxymethylene	particle	244	BASF	h)
polysulfone	particle	251	BASF	i)

Table S4 Materials placed on the Anodisc for sample RefC.

polymer	sample form	ATR spectrum	manufacturer	position on Filter (see Figure S3)
polylactic acid	foil 50 µm	177	Pütz GmbH + Co. Folien KG	a)
black rubber	particle	057	Gardena	b)
nitrile rubber	particle	145	Carl Roth GmbH	c)
polyethylene, oxidized	particle	014	Alroko	d)
silicone	particle	288	a2tec GmbH	e)
polychloroprene	particle	215	Scientific Polymer Products, Inc.	f) and g)
chitin	particle	038	Natural Sample	h)
polycaprolactone	particle	211	Abifor	i)
sand	particle	264	Merck	j)
animal fur	fibre	314	Faserinstitut Bremen	k)

Table S5 Materials placed on the Anodisc for sample RefD.

polymer	sample form	ATR spectrum	manufacturer	position on Filter (see Figure S4)
aramid	fibre	134	Dr. Dietrich Müller GmbH	a)
polyimide	foil 50 µm	133	Dr. Dietrich Müller GmbH	b)
ethylene vinyl alcohol / ethylene vinyl acetate copolymer (40%)	particle	076	Scientific Polymer Products, Inc.	c)
black rubber	particle	058	Gardena	d)
polyhydroxy butyric acid	particle	024	Biomer	e)
polybutadiene	particle	001	Scientific Polymer Products, Inc.	f)
polyetheretherketone	fibre	218	Faserinstitut Bremen	g)
polylactic acid	fibre	209	Faserinstitut Bremen	h)
ethylene vinyl alcohol / ethylene vinyl acetate copolymer (14%)	particle	074	Scientific Polymer Products, Inc.	i)

Table S6 Cluster categories for determination of the adaptable database design.

assigned category	cluster
1. very important	animal fur<br>polyamide<br>polycarbonate<br>polyethylene / rubber<br>polyethylene, oxidized<br>polyester / polyethylene terephthalate / polybutylene terephthalate<br>plant fibre<br>polypropylene<br>polystyrene / styrene-acrylonitrile<br>polyurethane / varnish<br>polyvinyl chloride<br>sand<br>silicone
2. important	acrylonitrile butadiene<br>aramid<br>cellulose acetate / -propionate / polyvinylacetate<br>chitin 1<br>ethylene vinyl acetate<br>ethylene vinyl alcohol / -acetate<br>polymethyl methacrylate<br>nitrile rubber<br>polyacetal / polyoxymethylene<br>polybutadiene<br>polycaprolactone<br>polychloroprene<br>polyetheretherketone<br>polyhydroxy butyric acid<br>polyimide<br>polylactic acid<br>polysulfone<br>rubber 1<br>rubber 3<br>silica gel 1
3. less important	algae 3<br>honeycomb 1<br>honeycomb 2<br>honeycomb 3<br>hydroxypropyl- /methyl cellulose<br>phenoxy resin<br>polyisoprene, chlorinated
4. not important	acrylonitrile butadiene styrene 1<br>acrylonitrile butadiene styrene 2<br>chitin 2<br>ethylene ethyl acrylate<br>ethylene (meth)acrylic acid<br>poly (2,4,6-tribromostyrene)<br>poly(2,6-dimethyl-p-phenylene oxide)<br>polyacrylamide, carboxyl modified<br>polyethylene glycol / -oxide<br>polyphenylene sulfide<br>polyvinylbutyral<br>polyvinylformal<br>polyvinylidene fluoride<br>polyvinylstearate<br>styrene maleic anhydride<br>styrene maleic anhydride partial methyl ester<br>vinyl chloride vinyl acetate<br>vinylidene chloride acrylonitrile

Table S7 Materials introduced as transmission FTIR data.

material	manufacturer	cluster number adaptable database design
polyethylene, high density	Orbita Film GmbH	1
polypropylene	Orbita Film GmbH	4
polycarbonate	Dr. Dietrich Müller GmbH	6
polyamide	mf-folien GmbH	7
polyethylene terephthalate	Pütz GmbH + Co. Folien KG	12
cellulose	Pütz GmbH + Co. Folien KG	14
polyetheretherketone	Faserinstitut Bremen	17
polylactic acid	Pütz GmbH + Co. Folien KG	21
ethylene vinyl alcohol / ethylene vinyl acetate copolymer (14%)	Scientific Polymer Products, Inc.	23
polyimide	Dr. Dietrich Müller GmbH	24
nitrile rubber	Carl Roth GmbH	10
polychloroprene	Scientific Polymer Products, Inc.	18
polycaprolactone	Abifor	22
Fiber jute	Faserinstitut Bremen	14
polysulfone	BASF	16
polyoxymethylene	BASF	25
cellulose acetate	Gizeh	9
acrylonitrile butadiene	Scientific Polymer Products, Inc.	27
polystyrene	INEOS Styrolution	5
polymethyl methacrylate	Plexiglas	12

Table S8 General color code for the different clusters.

cluster number adaptable database design	cluster name	RGB in HEX
1	polyethylene	AC001A
2	polyethylene oxidized	D51606
3	polyethylene-chlorinated	B64F0A
4	polypropylene	AE7A0D
5	polystyrene	A88A11
6	polycarbonate	7B7B1C
7	polyamide	485C1D
8	polyvinylchloride	259C4F
9	cellulose chemical modified	9FE8B9
10	nitrile rubber	CDF3DB
11	polyester	006F77
12	acrylates/polyurethanes/varnish	00A7B5
13	animal fur	C0C0C0
14	plant fibres	808080
15	sand	404040
16	polysulfone	1CEEFF
17	polyetheretherketone	BFFBFF
18	polychloroprene	0000AC
19	chitin	DFDFDF
20	polyisoprene chlorinated	4F4FFF

21	polylactic acid	8282FF
22	polycaprolactone	B7B7FF
23	ethylene-vinyl-acetate	9FCFFF
24	polyimide	AC5600
25	polyoxymethylene	FF8306
26	polybutadiene	FFCA95
27	acrylonitrile-butadiene	FFE6CC
28	rubber type 1	FA007A
29	rubber type 2	FF8AC5
30	charcoal	FFCAE4
31	coal	A4A400
32	rubber type 3	F4F400

Table S9 Result for the re-analysis of all assigned spectra for the sample RevEnv1 following the methodology described in literature [1].

cluster	number of spectra analyzed	certain assignnment	uncertain assignnment	mis assignnment
1	45	37	6	2
4	753	602	151	0
5	152	152	0	0
6	1	1	0	0
7	33	33	0	0
8	30	30	0	0
11	4	2	2	0
12	430	428	2	0
13	126	65	41	20
14	4520	3683	774	63
15	10	3	4	3
16	495	480	15	0
18	1	0	0	1
19	10	8	2	0
23	1	1	0	0
25	3	1	2	0
32	112	41	50	21

1. Primpke S, Lorenz C, Rascher-Friesenhausen R, Gerdts G. An automated approach for microplastics analysis using focal plane array (FPA) FTIR microscopy and image analysis. Analytical Methods. 2017;9(9):1499-511. doi:10.1039/c6ay02476a.
